# Construction of a High-Stability Electrochemical Immunosensor for Determination of Acetamiprid and Fipronil

**DOI:** 10.3390/foods15162813

**Published:** 2026-08-12

**Authors:** Nan Wang, Peng-Wei Xu, Zhi-Wei Zhang, Guo-Yuan Xiong, Zhen-Lin Xu, Jing-Nan Meng

**Affiliations:** 1 College of Food Science and Engineering, Anhui Science and Technology University, Chuzhou 239000, China; wn1825686@163.com (N.W.);; 2 Guangdong Provincial Key Laboratory of Food Quality and Safety, College of Food Science, South China Agricultural University, Guangzhou 510642, China; 3 Anhui Province Key Laboratory of Functional Agriculture and Functional Food, Anhui Science and Technology University, Chuzhou 239000, China

**Keywords:** electrochemical immunosensor, pesticide residues, acetamiprid, fipronil, food safety

## Abstract

Acetamiprid (ACE) and fipronil (FIP) are extensively applied broad-spectrum insecticides. They are recalcitrant to degradation and accumulate along food chains, creating prominent residual hazards to food security and ecological environments. Herein, a high-stability competitive electrochemical immunosensor was established for quantitative detection of ACE and FIP. Cyclic voltammetry (CV) and electrochemical impedance spectroscopy (EIS) characterized the layer-by-layer assembly of sensing interfaces, verifying the validity of competitive immune recognition. Under optimal conditions, the sensor exhibited linear ranges of 0.49–125.0 ng/mL for ACE and 0.49–62.5 ng/mL for FIP, with all determination coefficients (R^2^ > 0.99). The limits of detection (LODs) reached 0.33 ng/mL and 0.25 ng/mL, respectively. Spike-recovery detection on six fruit and vegetable matrices yielded recoveries of 84.63–100.80% with RSDs less than 7.8%, which confirmed excellent consistency between the immunosensor and standard LC-MS. This sensing platform combines fast response, high sensitivity, and superior stability, delivering a novel technical approach for rapid on-site screening of trace ACE and FIP residues in agricultural and environmental samples, and offering technical support for food safety supervision and pesticide risk assessment.

## 1. Introduction

Pesticides are indispensable agricultural production materials in modern intensive farming, which effectively control crop pests, reduce yield loss, and ensure food supply security [1,2]. Although novel pesticides such as nano-pesticides may serve as one of the key driving forces for advancing sustainable agriculture in the future [3], conventional pesticides are still widely applied in agricultural production at present. Acetamiprid (ACE) and fipronil (FIP) are two typical broad-spectrum insecticides widely used in agricultural pest prevention and control [4], which are commonly applied to control piercing–sucking and chewing pests in vegetables, fruits, grains, and cash crops [5,6]. Due to their good insecticidal effect and low cost, they have long-term market demand in agricultural production, but ACE and FIP are difficult to degrade, and can accumulate in the food chain step by step. Their improper use has led to potential residual risks in both the environment and agricultural products [7,8]. Although ACE and FIP differ greatly in chemical structure, mode of action, application specifications, and statutory maximum residue limits (MRLs), both insecticides are widely applied in vegetable and fruit cultivation. In actual agricultural practice, mixed or alternate application of neonicotinoid and phenylpyrazole pesticides frequently occurs, which creates a high possibility of co-occurrence of ACE and FIP residues in agricultural commodities. Therefore, it is necessary to establish independent rapid detection strategies suitable for the two representative pesticides, respectively, to satisfy diversified monitoring demands in food safety supervision. Against this background, developing reliable analytical approaches for ACE and FIP is of great practical significance to safeguard food safety and monitor environmental pollution.

At present, the reported detection technologies for ACE and FIP residues are diverse, mainly including instrumental analysis [9], spectral detection [10], immunoassay [11], and other categories [12], each with distinct application advantages and limitations. Instrumental analysis has the advantages of high accuracy, high sensitivity, and good selectivity and can realize qualitative and quantitative detection of trace pesticide residues and their metabolites, which are widely used in laboratory standard detection and supervision and inspection [13]. Spectral detection has the characteristics of fast detection and non-destructive analysis, which can realize rapid qualitative screening of pesticide residues [14]. Immunoassays boast simple operation and low cost, suitable for primary rapid screening [15]. However, these conventional approaches suffer from obvious drawbacks: chromatographic instruments are bulky and costly with time-consuming pre-treatment, many spectroscopic methods lack sufficient quantitative capacity, and most reported immunoassays display narrow linear ranges or unsatisfactory stability, which hinders their broader practical application. Electrochemical immunoassay combines the high specificity of antigen–antibody immune recognition reaction with the high sensitivity, fast response, and simple equipment of electrochemical detection technology [16,17], which effectively make up for the deficiencies of traditional detection methods.

In this study, high-stability competitive electrochemical immunosensors were constructed for separate quantitative detection of ACE and FIP residues. Rational optimization of the sensing interface and the competitive immune system was implemented to promote detection sensitivity, specificity, and operational stability. Distinct from many previously reported sensors with narrow linear working intervals, the developed platform achieves a wide detection range that covers the maximum residue limits specified in GB 2763-2026 [18], avoiding extra dilution of sample extracts during real-sample testing. The analytical performance was systematically validated via spike-recovery tests on fruit and vegetable matrices. This work provides an efficient, stable, and low-cost novel technical tool for monitoring trace ACE and FIP residues in agricultural products and offers technical support for food safety supervision and pesticide risk assessment.

## 2. Materials and Methods

### 2.1. Apparatus and Reagents

Cyclic voltammetry (CV), differential pulse voltammetry (DPV), and electrochemical impedance spectroscopy (EIS) analyses were carried out via the CHI660E electrochemical workstation, which was purchased from Chenhua Instrument Co., Ltd. (Shanghai, China). The three-electrode system consists of a glassy carbon electrode (GCE) with a 3 mm diameter as the working electrode, an Ag/AgCl electrode as the reference electrode, and a platinum (Pt) wire electrode as the auxiliary electrode. The GCE, Ag/AgCl, and Pt electrode were purchased from Yueci Electronic Technology Co., Ltd. (Shanghai, China).

The specific monoclonal antibodies (anti-ACE and anti-FIP) and coating antigens were purchased from Lvshiyuan Biotechnology Co., Ltd. (Shenzhen, China), and ACE and FIP standard solutions and their analogs were obtained from Tanmo Quality Inspection Technology Co., Ltd. (Changzhou, China). Chitosan, ethanol, potassium ferricyanide (K_3_[Fe(CN)_6_]), iron (II) cyanide potassium (K_4_[Fe(CN)_6_]•3H_2_O), Bovine albumin (BSA), and potassium chloride (KCl) were purchased from Sinopharm Group Chemical Reagent Co., Ltd. (Shanghai, China).

### 2.2. Electrochemical Analysis

The bare GCE was successively polished and ground with 0.3 µm and 0.05 µm of Al_2_O_3_ powder slurry. Then, it was rinsed with ultra-pure water, and subsequently thoroughly cleaned by ultrasonic treatment for 30 s using ethanol and ultra-pure water, respectively, until the cyclic voltammetry scan in a mixed solution of 5.0 mM Fe[(CN)_6_]^3−/4−^ and 0.1 M KCl was stable. It was then air-dried at room temperature and ready for use. On the pre-treated GCE, certain concentrations and volumes of chitosan solution, coating antigens, BSA, antibody, and the target molecule were successively and evenly dropped for modification. For each step, after the previous modified solution was completely dried, the electrode needed to be washed with phosphate-buffered saline (PBS, pH = 7.4) to remove the molecules that did not successfully incubate on the electrode surface, thereby reducing the influence on the experimental results. The electrochemical properties of different modified electrodes were tested using CV and EIS, and the sensitivity, reproducibility, stability, and specificity were evaluated by DPV. The parameter settings for electrochemical tests were as follows. In CV tests, the scan potential ranged from −0.4 V to 0.8 V, with a scan rate of 50 mV/s. In EIS tests, the frequency range was from 0.01 to 100 kHz, the amplitude was 5 mV, and the open-circuit voltage was 0.2 V. In DPV tests, the scan potential ranged from −0.2 V to 0.6 V, with a scan rate of 50 mV/s.

### 2.3. Fabrication of the Immunosensor

The pre-treated GCE was uniformly dropped with 5 μL (0.3%) chitosan, and incubated for drying to complete the primary modification. Then, 3 μL of ACE (1.0 μg/mL) or FIP (0.25 μg/mL) coating antigen was dropped onto the electrode. Next, 3 μL of BSA (1%, *w*/*v*) was added to the modified electrode and incubated for 20 min to block non-specific binding sites. Subsequently, 3 μL of the specific antibody for ACE (10.0 μg/mL) or FIP (6.0 μg/mL) was added to the electrode. Finally, 3 μL of ACE or FIP standard solutions with different concentrations was added. Each step of the modification process was carried out in a 37 °C oven. After each step was completed, all samples were gently rinsed with PBS and dried.

### 2.4. Detection of Real Samples

Fruits and vegetables are common food matrices contaminated with pesticide residues [19]. Zucchini, cauliflower, and grape were selected as real samples to evaluate the analytical performance of the immunosensor for ACE, while cowpea, cucumber, and apple were used for the FIP immunosensor. The selection of these matrices follows the maximum residue limits for pesticides in food in the China Food Safety Standard (GB 2763-2026). This standard sets specified MRLs for ACE and FIP on different agricultural commodities, and not all matrices possess regulatory MRLs for both analytes. Therefore, representative commodities with corresponding official MRLs for each pesticide were selected separately to fit practical food safety monitoring requirements. The spiked concentration gradients were also formulated according to the MRL requirements in GB 2763-2026. ACE was spiked at final concentrations of 100 ng/g, 200 ng/g, and 500 ng/g, whereas FIP was spiked at 1 ng/g, 10 ng/g, and 100 ng/g. Prior to the spiking experiment, blank fruit and vegetable samples were verified to be free of ACE and FIP residues. Hence, the native concentrations of ACE and FIP in all blank matrices were considered to be 0 ng/g.

The pre-treatment procedures for food samples were as follows. Briefly, each sample was homogenized, and 5 g of the homogenate was weighed out. Target pesticides were spiked into the corresponding homogenized samples to achieve the designated fortified concentrations on a sample mass basis. Subsequently, the mixtures were shaken for 2 min to achieve uniform dispersion of ACE and FIP. Then, the spiked sample was extracted with 25 mL acetonitrile to achieve a sufficient extraction efficiency of spiked ACE and FIP. Compared with many other organic solvents, acetonitrile can extract pesticides while minimizing co-extraction of interfering matrix substances such as lipids and pigments [20]. Afterward, 1 g sodium chloride and 4 g anhydrous magnesium sulfate were added to each homogenate. The mixture was vortexed thoroughly and centrifuged at 4500 rpm for 10 min to achieve phase separation. A 10 mL aliquot of the supernatant was transferred and dried under nitrogen gas. Finally, the residue was reconstituted with 2 mL PBS to maintain the same final concentrations as the spiked samples. All experimental data in this study were processed by Origin 2022 (OriginLab Corporation, Northampton, MA, USA). The molecular structures of ACE, FIP standards and their analogs were drawn using ChemDraw (PerkinElmer Informatics, Waltham, MA, USA).

## 3. Results and Discussion

### 3.1. Feasibility Analysis

**Figure 1:**
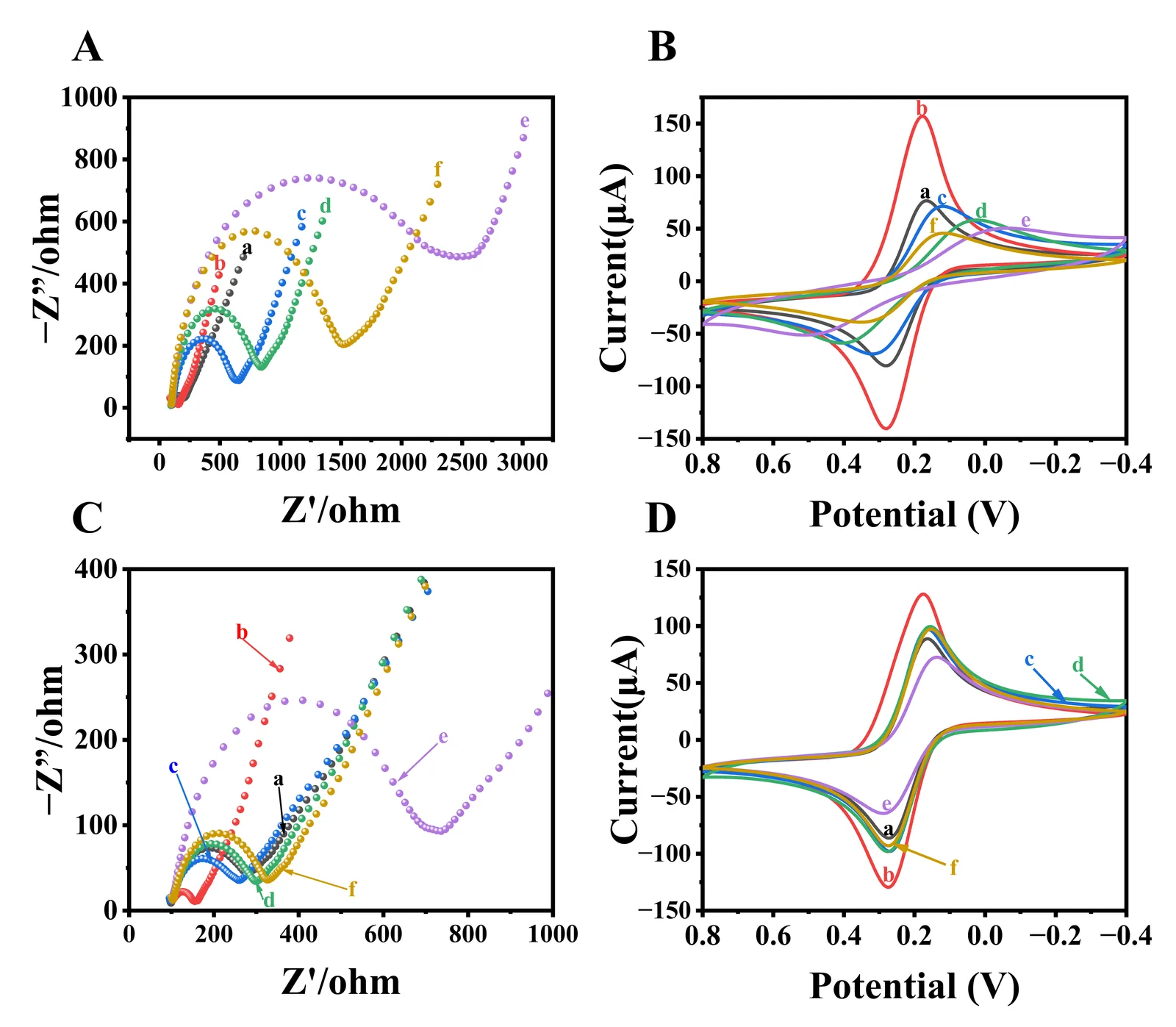
EIS (**A**) and CV (**B**) curves of the electrodes used for ACE detection. EIS (**C**) and CV (**D**) curves of the electrodes used for FIP detection. The lowercase letters in the picture represent the electrodes at different modification stages: a: the bare GCE, b: GCE/chitosan, c: GCE/chitosan/coating antigens, d: GCE/chitosan/coating antigens/BAS, e: GCE/chitosan/antigens/BAS/antibody, f: GCE/chitosan/coating antigens/BAS/antibody/target.

EIS and CV were utilized to characterize the interfacial electrochemical properties of the working electrode at each fabrication step, so as to systematically verify the feasibility of the proposed electrochemical competitive immunoassay for the detection of ACE and FIP. The electron transfer resistance (*Ret*), which reflects the diameter of the semicircle in impedance spectra [21], of electrodes at six successive modification stages were recorded and compared. As shown in Figure 1A, since the sensing interface of the bare electrode directly undergoes electron transfer, its *Ret* was relatively small (curve a) [22]. When the chitosan solution was drop-cast, chitosan, as an electrode modification material, improved the electrochemical performance of the electrode via multiple physicochemical mechanisms including electrostatic enrichment, accelerated interfacial electron transfer, and enlarged effective electrode area (curve b) [23]. As protein molecules of coated antigen, BSA, and antibody were sequentially immobilized onto the electrode surface, electron transfer was blocked, resulting in a prominent rise in Ret (curve c to curve e). Nevertheless, after ACE molecules competed with the coated antigen to occupy partial antibody binding sites, the diameter of the high-frequency (curve f) capacitive semicircle in the Nyquist plot shrank compared with curve e. This phenomenon indicated that the biomaterials modified on the electrode surface exert weaker obstruction against electron transport, accompanied by a reduction in electron transfer resistance. The variation trend of impedance was fully consistent with the anticipated immunorecognition process, which sufficiently verified the feasibility of the as-fabricated electrochemical immunosensor for ACE detection. The sensor can realize specific recognition and quantitative detection of ACE based on the competitive immunoassay principle. The trend of interfacial modification reflected by CV curves obtained at six successive electrode modification stages was completely consistent with the EIS results (Figure 1B). Figure 1C and Figure 1D correspond, respectively, to the EIS (C) and CV (D) curves of FIP for the six consecutive electrode modification steps. The interface impedance change trend shown in the EIS curve and the current change rule reflected in the CV curve are also consistent with the results obtained in the ACE detection system, which verify the feasibility of this immunosensor for the detection of the target pesticides.

### 3.2. Optimization of Parameters

**Figure 2:**
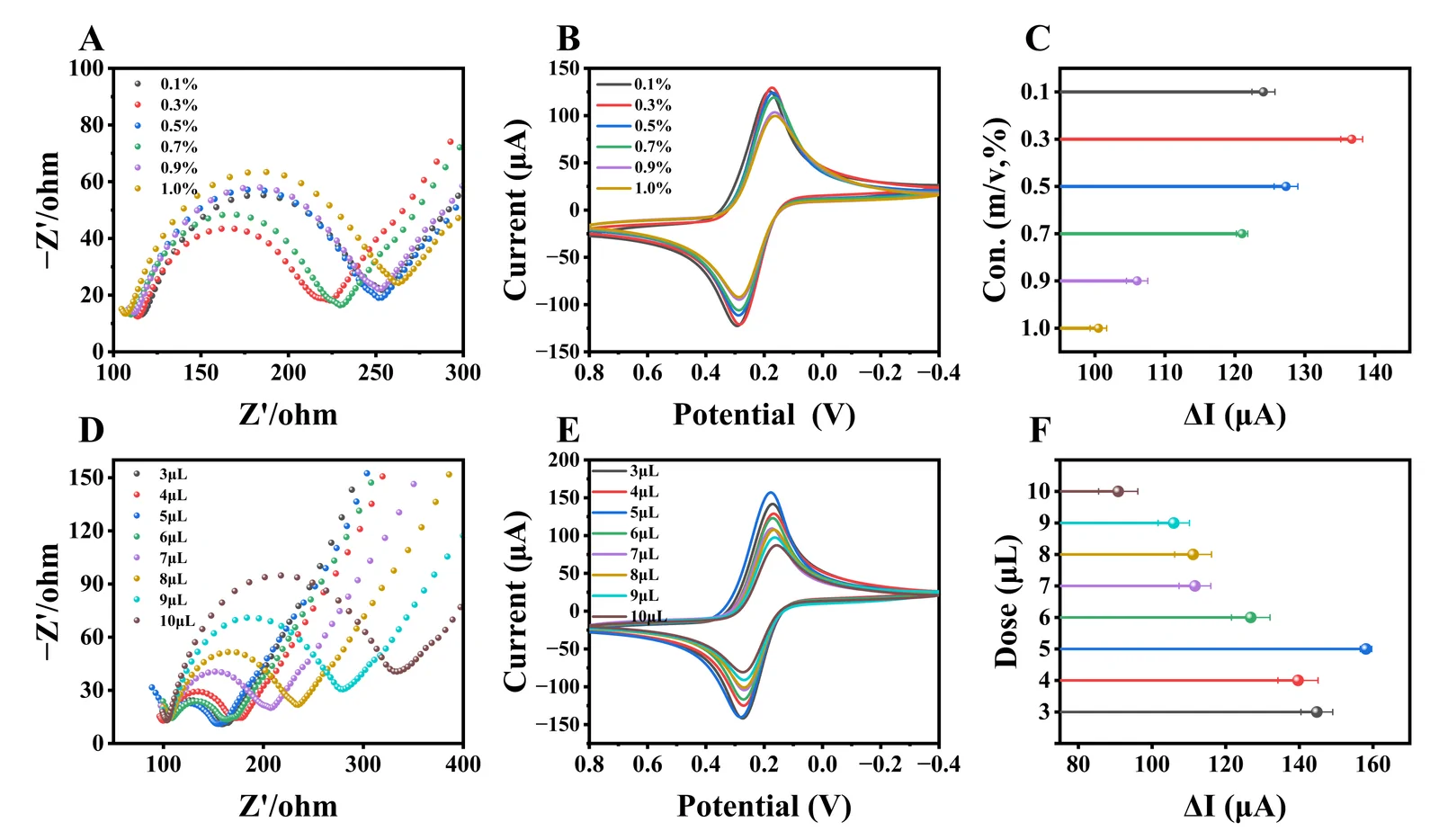
EIS plots (**A**) and CV curves (**B**) of electrodes modified with chitosan at different concentrations, and the effect of chitosan concentration on oxidation peak current (**C**). EIS plots (**D**) and CV curves (**E**) of electrodes modified with different dosages of chitosan, and the effect of chitosan dosage on oxidation peak current (**F**).

To optimize the detection performance, experimental parameters affecting the sensor performance were optimized in this study, including the concentration and dosage of chitosan, coated antigen concentration, blocking time, and target competitive incubation time. The optimization results of each parameter were evaluated based on the oxidation peak current of the electrode. Chitosan, a natural cationic polysaccharide abundant in amino (-NH_2_) and hydroxyl (-OH) groups [24], is one of the most widely used substrate modification materials for electrodes in immunosensors [25]. Its main functions include electrode interface modification, biological immobilization, signal amplification, and anti-interference [26]. Although chitosan itself is a non-conductive polymer, drop-casting forms a porous hydrophilic membrane on the electrode surface. The porous architecture enables the penetration and diffusion of redox probes, while protonated amino groups endow the film with a positive surface charge to electrostatically enrich negatively charged electroactive species. This electrostatic enrichment effect facilitates interfacial electron transfer and counteracts the inherent insulating property of chitosan. As shown in Figure 2A–C, when the dosage of chitosan was fixed at 3 μL, the amount of chitosan modified on the electrode surface increased with the rise in chitosan concentration, generating more positively charged amino groups (-NH_2_) that could enrich negatively charged molecules more efficiently. Meanwhile, the thickened film provided abundant additional reactive active sites. Consequently, the oxidation peak current increased as the chitosan concentration increased before reaching the optimal concentration of 0.3%. Nevertheless, excessively high chitosan concentration formed an overly thick and compact chitosan film, which not only hindered the diffusion of molecules toward the electrode surface but also increased the electron transfer resistance, ultimately resulting in a decreased current response. The drop-cast volume governs the thickness of the chitosan film formed on the electrode surface after drying. Only at the optimal dosage can the chitosan film supply sufficient active sites to efficiently enrich negatively charged substances in the solution, while avoiding excessive obstruction to mass diffusion and electron transfer, thus yielding the maximum current response (Figure 2D–F). Therefore, the optimal concentration of chitosan was determined to be 0.3%, and the optimal drop-cast volume was 5 μL.

To further obtain the optimal sensor parameters, the coating antigen volume was fixed at 3 μL, and the coating antigen concentration was adjusted in the range of 0.06–2.0 μg/mL. By evaluating its influence on the oxidation peak current, the optimal concentrations of coating antigens for ACE and FIP were determined to be 1.0 μg/mL (Figure S1A) and 0.25 μg/mL (Figure S1B), respectively. The volume of blocking solution BSA (1%, *w*/*v*) was fixed at 3 μL to evaluate the effect of blocking time on sensor performance. The results showed that both the ACE and FIP systems reached a stable signal at 20 min (Figure S2A,B). The antibody dosage was fixed at 3 μL (10 μg/mL for ACE, 6 μg/mL for FIP) and the concentration of the competitive antigen was set to 100 ng/mL to optimize the competitive incubation time, ranging from 5 min to 25 min. As illustrated in Figure S3A,B, the maximum inhibition rate, which was calculated according to Formula (1), was achieved after 10 min of competitive reaction for both ACE and FIP.

(1) $$\mathrm{Inhibition}\;\mathrm{rate}\;(\%)=\frac{I-I_{0}}{I_{0}}\times 100\%,$$

where *I*_0_ is the oxidation peak current without the target pesticide, and *I* is the oxidation peak current in the presence of the target pesticide.

### 3.3. Specific Analysis

**Figure 3:**
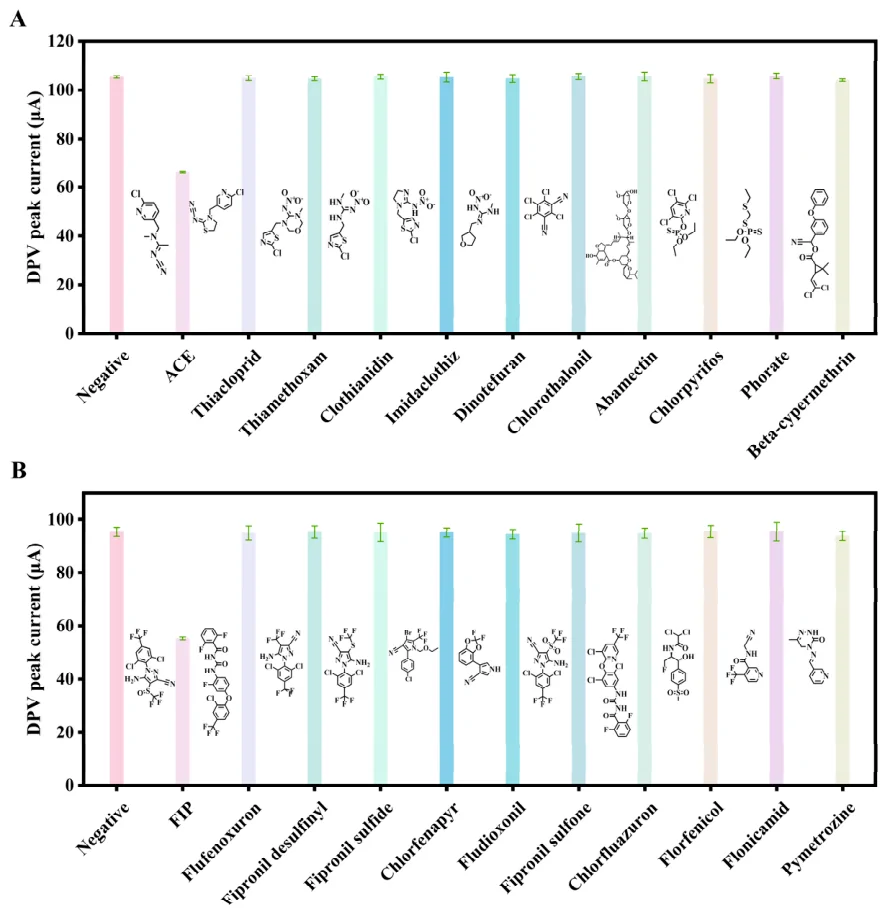
Specificity of the fabricated electrochemical immunosensors for ACE (**A**) and FIP (**B**). The concentration of both ACE and FIP was 100 ng/mL.

To evaluate the specificity of the fabricated electrochemical immunosensors for ACE and FIP, interference assays were carried out with ten structural analogs using DPV peak current as the response signal. The concentration of each interfering compound was set to 10 times that of the target analyte, while all other experimental conditions remained identical to those used for target detection. For the ACE immunosensor, the interfering substances included thiacloprid, thiamethoxam, clothianidin, imidaclothiz, dinotefuran, chlorothalonil, abamectin, chlorpyrifos, phorate, and beta-cypermethrin. For the FIP immunosensor, the interferences comprised flufenoxuron, FIP desulfinyl, FIP sulfide, chlorfenapyr, fludioxonil, FIP sulfone, chlorfluazuron, florfenicol, flonicamid, and pymetrozine. Figure 3A,B revealed that the current responses of interfering substances had no obvious difference from the blank control, demonstrating that these analogs exerted negligible interference on the detection of target analytes. Such results verified that the fabricated electrochemical immunosensors possessed excellent specific recognition performance for ACE and FIP, and could effectively discriminate target pesticides from other structurally similar interfering compounds.

### 3.4. Analytical Performance

**Figure 4:**
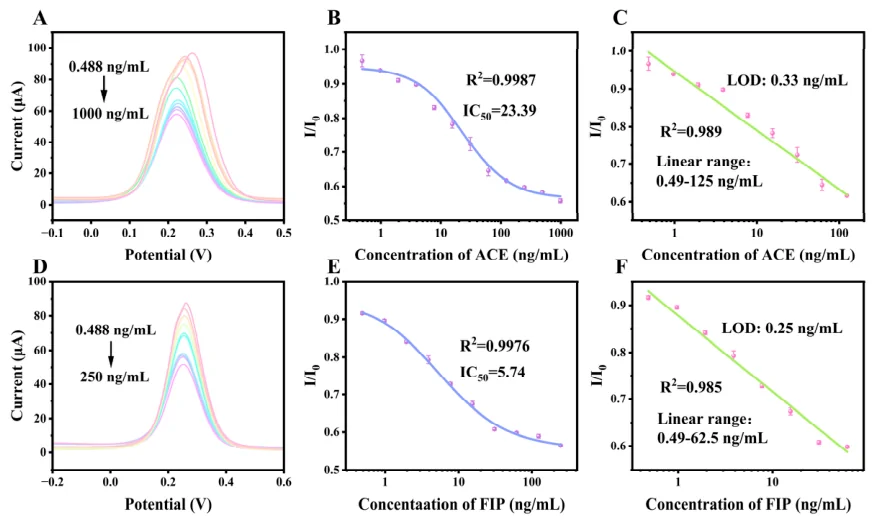
(**A**) DPV curves of the electrochemical immunosensor responding to the different concentrations of ACE. (**B**) Sigmoidal plot and (**C**) linear plot of DPV peak current vs. the ACE concentrations. (**D**) DPV curves of the electrochemical immunosensor responding to the different concentrations of FIP. (**E**) Sigmoidal plot and (**F**) linear plot of DPV peak current vs. the FIP concentrations.

Under the optimized experimental parameters, the fabricated electrochemical immunosensor generated typical DPV signals toward serially diluted ACE (0.49–1000 ng/mL) and FIP (0.49–250 ng/mL). Specifically, the DPV peak current difference decreased markedly with the increasing concentration of target analytes (Figure 4A,D). Within the tested concentration ranges, the correlations between the concentrations of the target (ACE and FIP) and corresponding peak current differences fit the five-parameter logistic (5PL) sigmoidal function embedded in Origin software. The coefficients of determination (R^2^) of the fitting equation for ACE and FIP were all higher than 0.99 (Figure 4B,E). The immunosensor exhibited half-maximal inhibitory concentrations (IC_50_) of 23.39 ng/mL and 5.74 ng/mL for ACE and FIP. Furthermore, linear regression analysis revealed that the immunosensor achieved linear detection ranges of 0.49–125 ng/mL for ACE and 0.49–62.5 ng/mL for FIP (Figure 4C,F). The corresponding limits of detection (LODs) were calculated to be 0.33 ng/mL and 0.25 ng/mL, respectively, where the LOD was calculated based on the 3σ/m criterion, where σ is the standard deviation of the blank and m is the slope of the calibration plot.

To further evaluate the analytical performance of the as-fabricated immunosensor, the reproducibility, repeatability, and storage stability of the electrodes were investigated separately using the DPV peak current as the response signal at fixed concentrations of ACE and FIP. Under identical experimental conditions, six parallel electrodes for ACE and FIP detection were each measured in triplicate to assess inter-electrode reproducibility, while a single electrode was subjected to six consecutive cyclic measurements to evaluate intra-electrode repeatability. For storage stability assessment, three modified electrodes were preserved in a refrigerator at 4 °C and tested every two days throughout the storage period. All relative standard deviation (RSD) values obtained from the above tests were less than 5% (Figure S4A,B), demonstrating the favorable reproducibility, repeatability, and storage stability of this electrochemical immunosensor.

### 3.5. Performance of the Sensor

**Figure 5:**
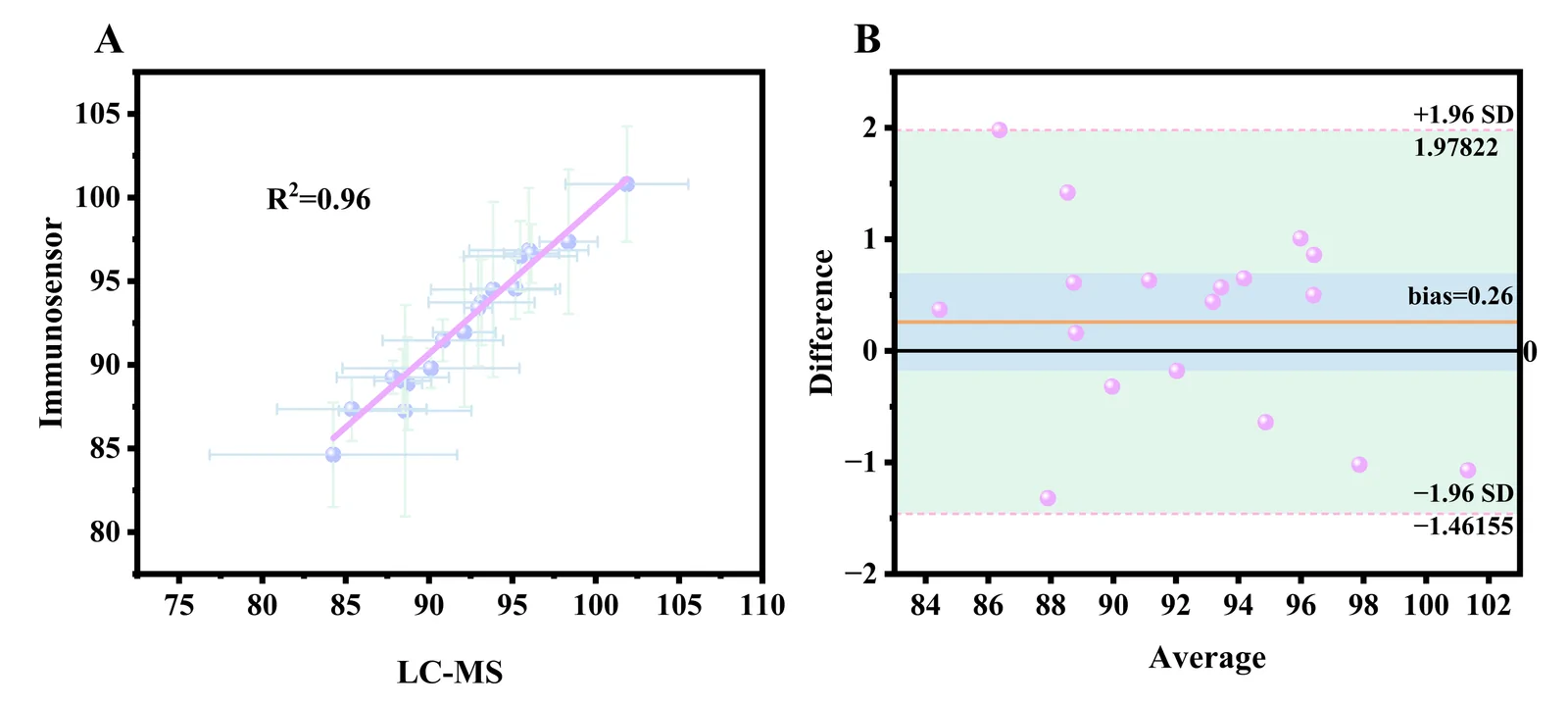
Evaluation of the consistency between the immunosensor and LC-MS by linear scatter plot fitting (**A**) and Bland–Altman (**B**).

The analytical performance of the fabricated electrochemical immunosensor was systematically verified via spike-and-recovery tests in six real food matrices, including zucchini, cauliflower, and grape for ACE detection, and cowpea, cucumber, and apple for FIP quantification. Three fortified concentration levels were set for each matrix, with three parallel samples prepared per concentration. The average recoveries of ACE and FIP measured by the immunosensor ranged from 84.63% to 100.80%, and all relative standard deviations (RSDs) were below 7.8%, demonstrating acceptable accuracy and favorable intra-batch precision (Table 1). To further validate the reliability of the proposed sensing strategy, LC-MS was adopted as the reference standard method for parallel detection of all spiked samples (Table S1). The linear scatter plot fitting (Figure 5A) and Bland–Altman (Figure 5B) proved that the consistency between the immunosensor and LC-MS results exceeded 95% for all test groups, which confirmed the outstanding practicability and satisfactory accuracy of the immunosensor for pesticide residue screening in complex vegetable and fruit samples. Collectively, this electrochemical immunosensor exhibited reliable recovery, low measurement deviation, and high consistency with instrumental chromatography, showing great potential for rapid on-site detection of ACE and FIP in agricultural products.

To further evaluate the performance of the fabricated electrochemical immunosensor, a comprehensive comparison between the detection strategy established in this work and previously reported approaches is summarized in Table 2. The proposed strategy integrates the merits of a broad detection range and competitive sensitivity. Its linear range can cover the MRLs of ACE and FIP in various fruits and vegetables, eliminating the need to dilute sample extracts during actual detection and simplifying pre-treatment operations. Compared with HPLC-MS/MS, this electrochemical platform enables rapid measurement with portable equipment and lower testing costs. Nevertheless, several reported SERS and lateral flow immunoassays achieve lower LODs for FIP. Independent sensors were constructed for the two analytes rather than realizing simultaneous detection, and the current pre-treatment relies on acetonitrile extractant. Further optimization of sensitivity, multiplex detection capacity, and greener sample preparation should be pursued in follow-up research.

## 4. Conclusions

In this study, a high-stability electrochemical competitive immunosensor was successfully fabricated for the quantitative detection of ACE and FIP pesticide residues. Chitosan modification effectively improved the electrode interfacial electrochemical performance by providing abundant active sites and promoting electron transfer, laying a reliable foundation for the immobilization of coating antigens, blocking proteins, and specific monoclonal antibodies. Anti-interference tests verified that the sensor could specifically recognize target pesticides without obvious response to structurally similar interfering substances. Under optimal experimental conditions, the sensor achieved reliable linear detection ranges of 0.49–125.0 ng/mL for ACE and 0.49–62.5 ng/mL for FIP, with low LODs of 0.33 ng/mL and 0.25 ng/mL, respectively. The constructed immunosensor exhibited favorable analytical performance, including a wide linear range, a low detection limit, excellent repeatability, inter-electrode reproducibility, and long-term storage stability. Actual sample detection on zucchini, cauliflower, grape, cowpea, cucumber, and apple showed satisfactory recovery (84.63–100.80%) and precision (RSD < 7.8%), and the test results were highly consistent with the reference LC-MS method, proving its reliability in complex food matrix detection. In summary, the developed sensing platform possesses great application potential for monitoring of ACE and FIP residues in agricultural products, and can provide effective technical support for routine food safety supervision and environmental pollution risk evaluation. Subsequent research can further expand the sensor’s multiplex detection capability for more pesticide varieties and realize integrated portable sensing devices to better meet the demands of field rapid detection.

## Figures and Tables

**Table 1:** ACE and FIP detection in real samples by the electrochemical immunosensor.

Samples	Spiked Final Concentrations (ng/g)	Detection Concentrations (ng/g)	Recovery (%)	RSD (%)
Zucchini	100	93.73 ± 2.09	93.73	2.74
	200	201.59 ± 5.64	100.80	3.43
	500	472.77 ± 7.39	94.55	1.91
Cauliflower	100	96.49 ± 1.72	96.49	2.18
	200	177.76 ± 4.50	88.88	3.10
	500	448.98 ± 0.83	89.80	1.32
Grape	100	91.94 ± 3.65	91.94	4.86
	200	174.70 ± 3.11	87.35	2.18
	500	484.24 ± 15.21	96.85	3.85
Cowpea	1	0.95 ± 0.04	94.50	5.54
	10	9.74 ± 0.35	97.36	4.43
	100	91.46 ± 1.02	91.46	1.37
Cucumber	1	0.85 ± 0.03	84.63	3.68
	10	8.73 ± 0.52	87.25	7.24
	100	96.65 ± 1.43	96.65	1.81
Apple	1	0.93 ± 0.03	93.40	3.73
	10	8.90 ± 0.15	89.04	2.13
	100	89.25 ± 0.80	89.25	1.10

**Table 2:** Comparison of the analytical performance of different assays for ACE and FIP.

**Target**	Detection Method	Detection Range (ng/mL or ng/g)	LOD (ng/mL or ng/g)	Recovery (%)	CV (%)	Ref.
ACE	Fluorescent molecularly imprinted method	20.0–800.0	8.30	89.6–97.9	<2.9	[27]
ACE	Electrochemiluminescence	1.1–111.3	/	97.0–104.3	<3.7	[28]
ACE	Surface-enhanced Raman scattering	500.0–50,000.0	140.00	80.5–105.5	<11.7	[29]
ACE	Indirect competitive chemiluminescence enzyme immunoassay	0.7–96.3	0.70	82.7–112.2	<9.2	[30]
ACE	HPLC-MS/MS	17.3–500.0	17.00	85.9–103.4	<7.7	[31]
FIP	Surface-enhanced Raman scattering lateral flow immunoassay	0.006–4.0	0.005	97.0–117.0	<11.7	[32]
FIP	One-step enzyme-linked immunosorbent assay	0.02–20.0	/	76.7–93.7	<12.1	[33]
FIP	Incorporated molecularly imprinted polymer sensor	0.4–2.6	0.13	96.2–97.8	<4.0	[34]
FIP	Time-resolved fluorescent immunochromatographic assay	/	0.86	95.5–112.6	<10.2	[35]
FIP	HPLC-MS/MS	0.013–100.0	0.04	89.9–100.3	<5.1	[36]
ACE	Electrochemical competitive immunosensor	0.5–125.0	0.33	87.3–100.8	<4.9	This work
FIP		0.5–62.5	0.25	84.6–97.3	<7.3	

## Data Availability

The original contributions presented in the study are included in the article/Supplementary Materials. Further inquiries can be directed to the corresponding authors.

## Supplementary Materials

The following supporting information can be downloaded at: https://www.mdpi.com/article/10.3390/foods15162813/s1, Figure S1: Effect of concentrations of ACE (A) and FIP (B) coating antigens on the oxidation peak current; Figure S2: Effect of blocking time on the oxidation peak current: ACE (A) and FIP (B); Figure S3: Effect of competitive incubation time on the inhibition rate of ACE (A) and FIP (B); Figure S4: The reproducibility, repeatability, and storage stability of the electrodes; Table S1: ACE and FIP detection in real samples by LC-MS.
