# Supplementary material for: Construction of a High-Stability Electrochemical Immunosensor for Determination of Acetamiprid and Fipronil
Source: Foods. 2026 Aug 12;15(16):2813. doi: 10.3390/foods15162813 (PMC13512751; doi:10.3390/foods15162813)
Supplement: Supplementary file 1 [file foods-15-02813-s001.zip › foods-4467943-supplementary.pdf]

Construction of a High-Stability Electrochemical Immunosensor for Determination of Acetamiprid and Fipronil

Table S1. ACE and FIP detection in real samples by LC-MS.

Samples	Spiked final concentrations (ng/g)	Detection concentrations (ng/g)	Recovery (%)	RSD (%)
Zucchini	100	93.16±3.36	93.16	3.61
	200	203.75±6.36	101.87	3.12
	500	475.95±18.41	95.19	3.87
Cauliflower	100	95.48±2.68	95.48	2.8
	200	177.45±6.81	88.72	3.84
	500	450.62±4.36	90.12	0.97
Grape	100	92.12±5.31	92.12	5.77
	200	170.75±3.76	85.37	2.21
	500	479.95±22.42	95.99	4.67
Cowpea	1	0.94±0.04	93.85	3.8
	10	9.84±0.37	98.38	3.8
	100	90.83±1.74	90.83	1.92
Cucumber	1	0.84±0.04	84.26	4.31
	10	8.86±0.74	88.57	8.39
	100	96.15±3.98	96.15	4.13
Apple	1	0.93±0.02	92.96	1.78
	10	8.84±0.08	88.43	0.95
	100	87.83±1.71	87.83	1.94

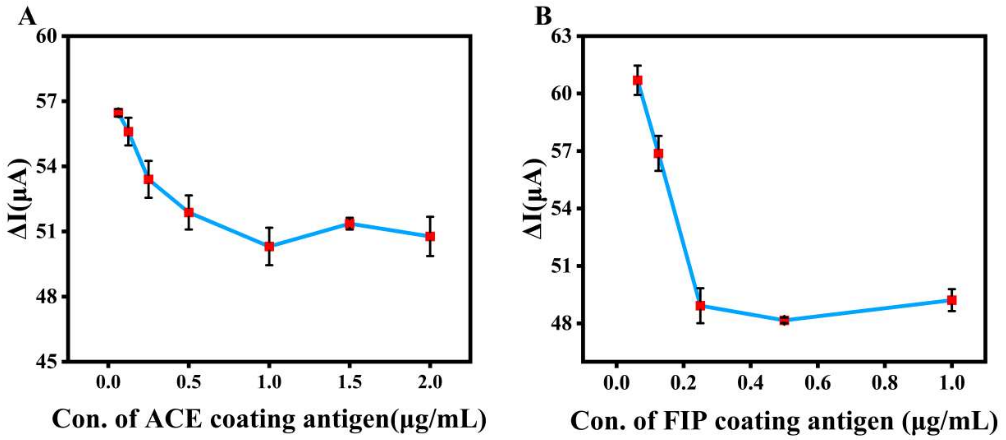

Figure S1. Effect of concentrations of ACE (A) and FIP (B) coating antigens on the oxidation peak current.

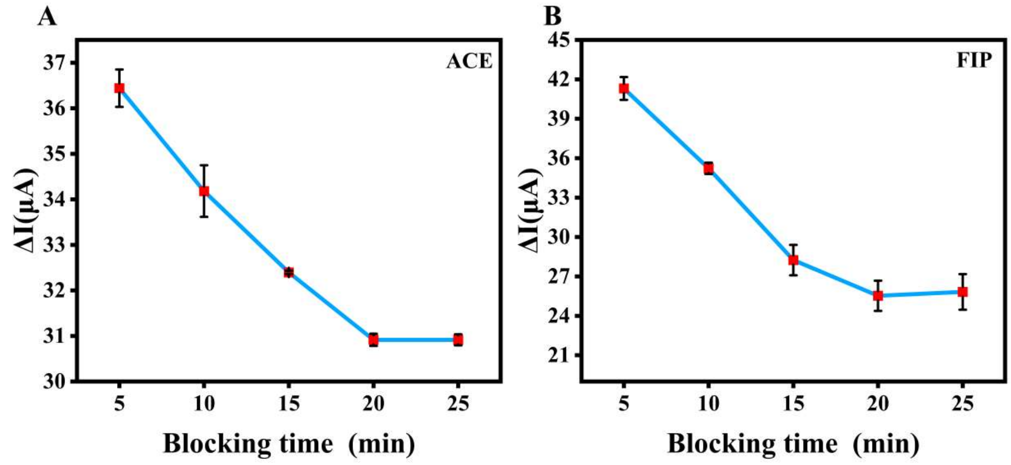

Figure S2. Effect of blocking time on the oxidation peak current: ACE (A) and FIP (B).

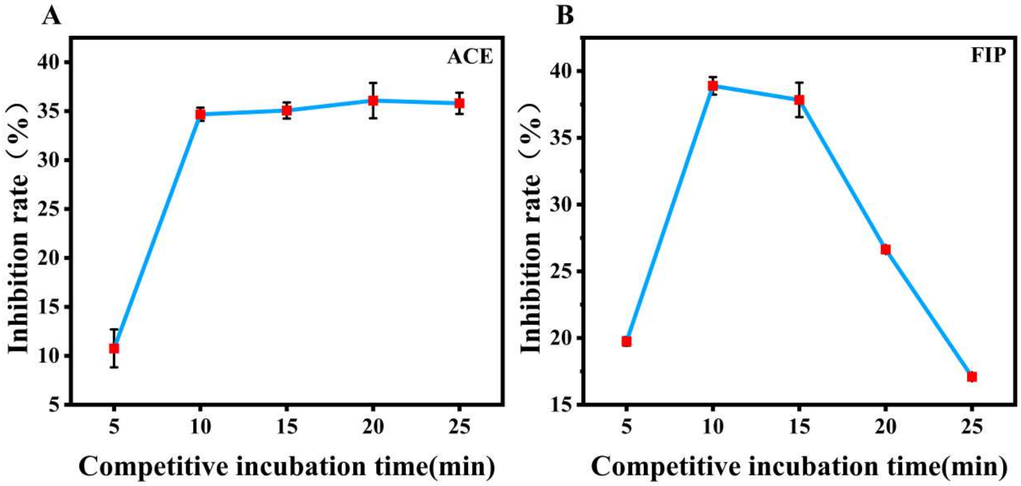

Figure S3. Effect of competitive incubation time on the inhibition rate of ACE (A) and FIP (B).

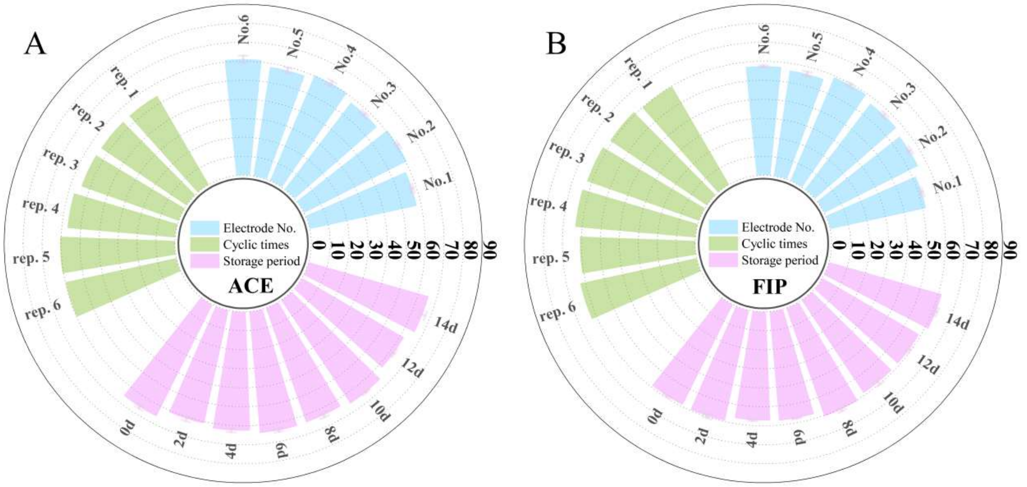

Figure S4. The reproducibility, repeatability, and storage stability of the electrodes.
